# Exercise Training in Patients with Heart Failure: From Pathophysiology to Exercise Prescription

**DOI:** 10.31083/j.rcm2304144

**Published:** 2022-04-12

**Authors:** Gianluigi Cuomo, Anna Di Lorenzo, Anna Tramontano, Francesca Paola Iannone, Andrea D’Angelo, Rita Pezzella, Crescenzo Testa, Alessandro Parlato, Pasquale Merone, Mario Pacileo, Antonello D’Andrea, Giuseppe Cudemo, Elio Venturini, Gabriella Iannuzzo, Carlo Vigorito, Francesco Giallauria

**Affiliations:** ^1^Department of Translational Medical Sciences, “Federico II" University of Naples, 80131 Naples, Italy; ^2^Division of Cardiology/UTIC, “Umberto I" Hospital, Nocera Inferiore (ASL Salerno), 84014 Nocera Inferiore (SA), Italy; ^3^Cardiac Rehabilitation Unit, Azienda USL Toscana Nord-Ovest, Cecina Civil Hospital, 57023 LI Cecina, Italy; ^4^Department of Clinical Medicine and Surgery, “Federico II" University of Naples, 80131 Naples, Italy

**Keywords:** heart failure, preserved ejection fraction, exercise training, cardiac rehabilitation, continuous training, interval training, strength training, respiratory training, inspiratory muscle training, functional electrical stimulation, mortality, elderly, frailty, COVID-19

## Abstract

Heart failure (HF) is a chronic, progressive, and inexorable syndrome affecting worldwide billion of patients (equally distributed among men and women), with prevalence estimate of 1–3% in developed countries. HF leads to enormous direct and indirect costs, and because of ageing population, the total number of HF patients keep rising, approximately 10% in patients >65 years old. Exercise training (ET) is widely recognized as an evidence-based adjunct treatment modality for patients with HF, and growing evidence is emerging among elderly patients with HF. We used relevant data from literature search (PubMed, Medline, EMBASE) highlighting the epidemiology of HF; focusing on central and peripheral mechanisms underlying the beneficial effect of ET in HF patients; and on frail HF elderly patients undergoing ET. Since many Countries ordered a lockdown in early stages pandemic trying to limit infections, COVID-19 pandemic, and its limitation to exercise-based cardiac rehabilitation operativity was also discussed. ET exerts both central and peripheral adaptations that clinically translate into anti-remodeling effects, increased functional capacity and reduced morbidity and mortality. Ideally, ET programs should be prescribed in a patient-tailored approach, particularly in frail elderly patients with HF. In conclusion, given the complexity of HF syndrome, combining, and tailoring different ET modalities is mandatory. A procedural algorithm according to patient’s baseline clinical characteristics [i.e., functional capacity, comorbidity, frailty status (muscle strength, balance, usual daily activities, hearing and vision impairment, sarcopenia, and inability to actively exercise), logistics, individual preferences and goals] has been proposed. Increasing long-term adherence and reaching the frailest patients are challenging goals for future initiatives in the field.

## 1. Introduction

Heart failure (HF) is a heterogeneous syndrome, which presents often non-specific symptoms and sign at the onset, but life-limiting with the disease progression [1]. About 64.3 million people are living with heart failure worldwide (equally distributed between men and women), with prevalence estimated at 1–3% in developed countries, but it grows to approximately 10% in people >65 years old [2]. Prevalence in Asia [3] and Latin America seems to be similar to Western countries: conversely, results difficult estimating prevalence in Africa due to scarce literature [2, 4]. Furthermore, this results enormous direct and indirect costs, estimated about $108 billion per annum worldwide in 2012 [5]. However, because of ageing population, the total number of HF patients keep rising [6].

Notably, HF strongly impacts on disability and is a major determinant of frailty: it has been assessed that 8.9% of patients have extreme disability and 30.3% have severe disability in life activities, while 53.3% of patients have moderate-severe disability in participation in society [7, 8, 9]. HF has negative impact on QoL similarly to other conditions (i.e., Parkinson’s disease), even though on optimal medical therapy: about 70% of patients suffer from pain and discomfort, and half of patients experience anxiety and depression [10].

HF untreated symptoms, in addition to effects on quality of life (QoL), increase hospitalizations, emergency department visits, and long-term mortality [11, 12]. In fact, despite advances in both pharmacological and non-pharmacological therapeutic strategies for HF, either with reduced or preserved ejection fraction (HFrEF and HFpEF, respectively), mortality and morbidity still remain elevated [13, 14].

In HF patients, structured moderate-intensity continuous exercise training is strongly recommended (Class I recommendation, level of evidence A) in order to improve symptoms relief, functional capacity and QoL and reduce hospitalization [15].

The reduction of hospitalization has been clearly documented for HF patients undergoing exercise-based cardiac rehabilitation [16, 17, 18]; interestingly, a recent study found that the acute-phase initiation of CR was associated with lower in-hospital mortality (odds ratio [OR] 0.76, 95% confidence interval [CI] 0.73–0.80), shorter hospital stays and lower incidence of 30-day readmission due to HF [19]. In addition, in a cohort of 190 elderly patients hospitalized for HF, Kono *et al*. [20] showed that early mobilization within 3 days from admission exert reduction of cardiac events in 1400 days follow-up from discharge compared to mobilization from 4th day of admission. Interestingly, early mobilization was shown as independent predictor of re-hospitalization.

However, few HF patients are referred to structured training program; and a standardized training protocol suitable for all patients has not yet been validated [21]. In HF patients, different types of training have been tested: continuous moderate training (MCT) [22], high intensity interval training (HIIT) [23] and resistance or strength training (RST) [24], alone or in combination (i.e., ARIS (Aerobic, Resistance, InSpiratory Training OutcomeS) protocol [25].

The European Society of Cardiology Guidelines recommend regular aerobic exercise in HF patients to improve functional capacity and symptoms and to reduce risk of hospitalization [15]. Similarly, Canadian Cardiovascular Society Guidelines for the Management of Heart Failure in 2017 recommended aerobic exercises in stable HF patients to improve exercise capacity [26], while American College of Cardiology Foundation/American Heart Association guidelines in 2013 recognized only exercise training effects on quality of life for these patients [27]. Nevertheless, only about half of patients for whom it would be indicated are enrolled in training protocols [28] and referral rate is quite scarce in some region.

This review discusses recent evidence on the effect of exercise training in patients with HF (HFrEF and HFpEF), moving from pathophysiology to exercise prescription.

## 2. Pathophysiological Effects of Exercise Training

Effects of exercise training on central cardiac and peripheral mechanisms have long been investigated. At the onset of exercise, cardiac output (the product of heart rate and stroke volume) may increase from ∼5 L/min at rest to ∼15 L/min in young (20–40 years old) females and ∼20 L/min in young males [29]. At early stages of exercise, rise in heart rate is the main cause of the increase in cardiac output, but the maximum heart rate may decrease during maximal exercise with training [30]. Therefore, the large increase in cardiac output after exercise training is due to a larger stroke volume.

One year of progressive endurance exercise training has shown to increase LV mass and LV end-diastolic diameters (LVEDD) in sedentary subjects. In left ventricle, the initial effect during the first 6–9 months is a concentric remodeling depending on the duration and intensity of exercise, while in the right ventricle an eccentric remodeling was seen in response to endurance training [31].

About peripheral mechanisms, exercise training showed to reduce the effects of hyperactivation of the sympathetic nervous system [32].

Higher levels of circulating catecholamines have been detected in patients with heart failure, due to dysregulation of the sino-aortic and cardiac baroceptor; this mechanism results in rising of noradrenalin circulating level [33], which may aggravate myocardial ischemia and cause arrhythmias [34].

Furthermore, ET has been shown to effect on vagal stimulation improving heart rate recovery (HRR), which is fall in heart rate during first minute after exercise and is correlated with long term prognosis in patients with HF [35, 36, 37, 38].

In patients with HF, exercise training could stimulate favorable left atrial [39] and left ventricle reverse remodeling after myocardial infarction decreasing circulating catecholamine and natriuretic peptide levels [40, 41, 42, 43].

Moreover, exercise training has anti-inflammatory and antioxidative effects, reducing proinflammatory cytokines concentration in skeletal muscle and increasing antioxidative molecules production [44, 45, 46, 47, 48, 49].

In addition, the above-mentioned effects contribute to peripheral vasodilation, improving endothelial function through nitric oxide (NO) production [50, 51].

Furthermore, exercise training plays a key role on cardiopulmonary efficiency, considering that dyspnea is one of main symptom of HF. Cardiopulmonary exercise testing (CPET) is the gold-standard method for measuring maximum exercise capacity and cardiorespiratory fitness. Oxygen uptake and ventilatory patterns obtained during the submaximal portion of CPET have strong relationship to prognosis in HF patients [52]. Several studies assessed relationship between lower cardiorespiratory fitness and risk of developing coronary artery disease and heart failure in older age [53, 54].

Moreover, low cardiorespiratory fitness, assessed by treadmill test, in young adulthood is associated with smaller left ventricle size; in addition, greater cardiorespiratory fitness decline with aging should indicate higher risk of developing LV dysfunction [55].

Exercise training could improve ${\mathrm{VO}}_{\text{2peak}}$ both in patients with HF with reduced left ventricular ejection fraction (HFrEF) and in patients with HF with preserved left ventricular ejection fraction (HFpEF), offering a further therapeutic option especially in the latter [56, 57]. Notably, it is crucial that exercise training should be performed life-long after acute event for maintaining long-term cardiovascular fitness [39].

**Fig. 1.**
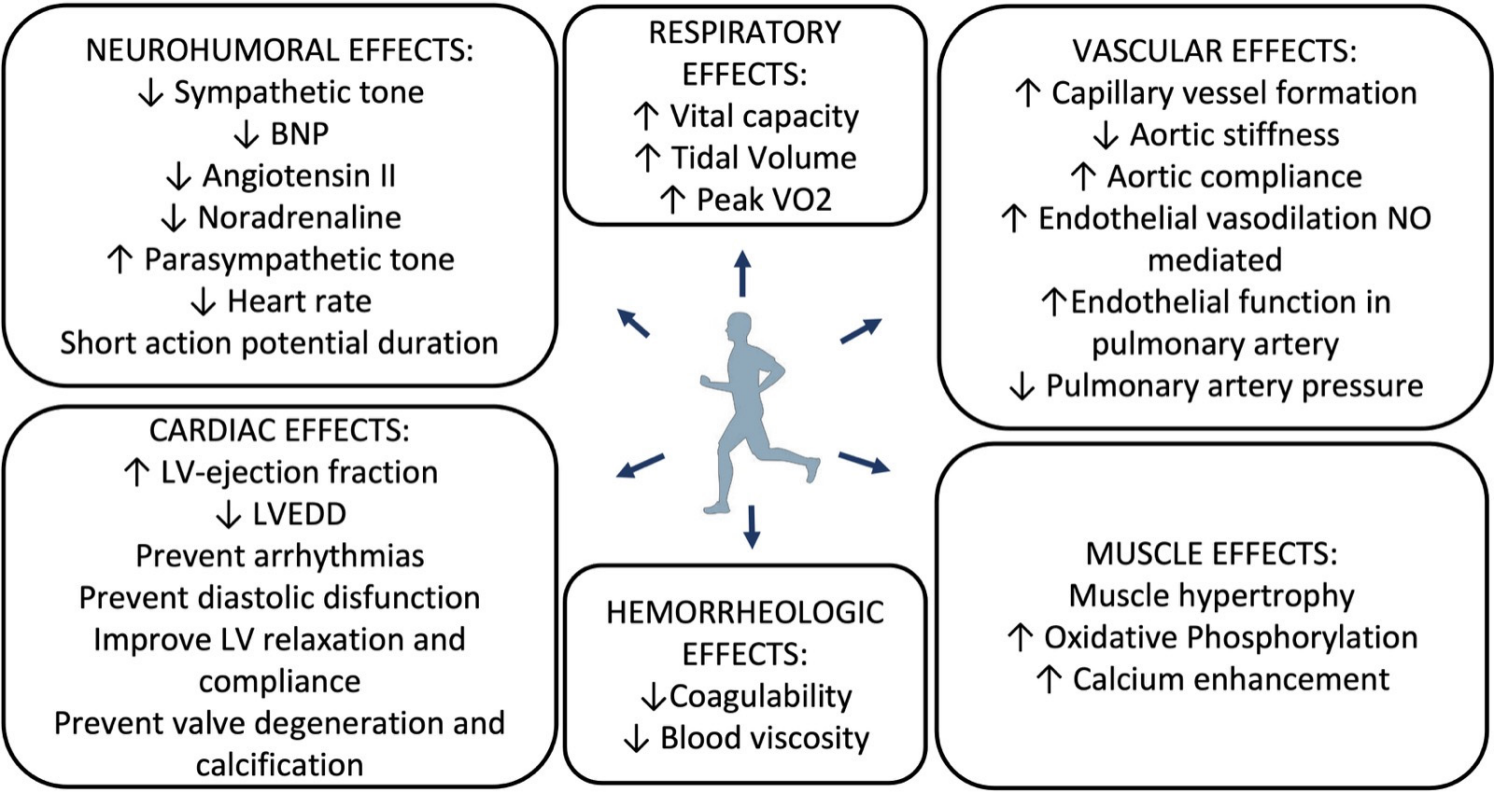
**Effects of exercise training in Heart failure**. BNP, brain natriuretic peptide; LV, left ventricular; LVEDD, left ventricular end diastolic diameter; NO, nitric oxide; peakVO2, peak oxygen consumption.

The main effects of exercise training in heart failure are summarized in Fig. 1.

## 3. Exercise Prescription in Heart Failure with Reduced Ejection Fraction (HFrEF)

Although most recent European Society of Cardiology guidelines recommend regular aerobic exercise in HF patients to improve functional capacity and symptoms and to reduce risk of hospitalization [15], only about half of patients for whom it would be indicated are enrolled in training protocols [28].

However, an exercise training program is only recommended in stable patients, in NYHA class II–III, undergoing optimal medical treatment, while it is contraindicated in a number of cardiac (first 2 days after acute coronary syndrome, untreated life-threatening cardiac arrhythmias, uncontrolled hypertension, acute heart failure, acute myocarditis and pericarditis, symptomatic aortic stenosis, severe hypertrophic obstructive cardiomyopathy) and non-cardiac diseases (acute systemic illness, uncontrolled diabetes mellitus, thrombophlebitis, severe COPD) [58].

Nevertheless, to date there are still no standardized training protocols. Several studies investigated different exercise training types, methods, and settings, to identify pathophysiological effects and benefits of various types of intervention. Anyway, it is recommended to carry out submaximal exercise test (6-Minute Walking Test or CPET) to evaluate exercise capacity and determine training intensity before starting any training protocol.

Six-Minute Walking Test (6MWT) is an easy to perform and widely used test, which may provide reliable information about HF prognosis and patient capacity to perform daily activities, but it suffers from physician ability, place where it is performed and patient condition [59].

Cardiopulmonary exercise testing (CPET) has been established to be safe in HF patients [60] and it is considered gold standard to assess exercise capacity and to determine exercise training intensity, measuring directly O2 consumed during exercise until this peak (${\mathrm{VO}}_{\text{2peak}}$) and providing an estimate of transition from aerobic to anaerobic metabolism, the ventilatory anaerobic threshold (VAT) [58, 61].

The first and more investigated form of training is endurance aerobic training or moderate continuous training (MCT) [22]. This modality can be performed by cycling or treadmill, without reaching maximum effort; after estimating exercise intensity through ${\mathrm{VO}}_{\text{2peak}}$ measurement, it is recommended to start at low intensity (about 5–10 minutes twice a week) and then increase according to patient’s tolerance (up to 20–60 minutes on 3–5 days a week) [58].

In HF-ACTION trial, continuous moderate training showed, after adjustment for highly prognostic predictors of the primary endpoint, a modest significant reduction for mortality and hospitalization in HF patients (HR = 0.85 for cardiovascular mortality or HF hospitalization; 95% CI, 0.74–0.99; *p* = 0.03) [22].

Interval training (IT) is based on short bouts alternating with recovery phases, using treadmill or electrically braked cycle. According to patient’s clinical features, two different programs are possible: high intensity interval training (HIIT) includes few (about three or four) hard work phases (3–4 minutes) performed at 90–95% of maximal exercise capacity, interspersed with recovery phases (3 minutes) performed at low or no workload; the whole is preceded by a warm-up and followed by a cool-down phase; low intensity interval training (LIT) consists in 15 minutes exercise alternating hard (about 30 seconds at 50% of achieved power output) and recovery (about 60 seconds) phases, and intensity should be increased accord to patient’s exercise conditioning (until 30 minutes training session).

Several studies in recent times compared MCT and HIIT [23, 62, 63, 64, 65], without reaching univocal results; although HIIT appears to be more effective than MCT in improving left ventricular function, possibly due to challenge on heart’s pumping ability caused by short bouts of exercise, recently SMARTEX-HF randomized multicenter trial by Ellingsen *et al*. [66] showed that HIIT was not superior to MCT in improving left ventricular end-diastolic diameter and ${\mathrm{VO}}_{\text{2peak}}$.

Resistance or strength training (RST) is based on muscle contraction exercises against specific resistances, with aim of increasing muscle strength and endurance [24]. Therefore, it is an anaerobic exercise, widely used to prevent wasting syndrome; in this instance, more subjective parameters are used to determine exercise intensity, such as % of one repetition (% 1-RM, i.e., maximum weight that can be lifted only once [25]) or Borg scale [67].

Due to possible negative effects on remodeling and ventricular overload and the poor evidence of efficacy, RST has been underused for long [68, 69, 70]; however, its use has recently been increased in association with aerobic endurance and interval training, showing additional benefits on respiratory parameters (particularly ${\mathrm{VO}}_{\text{2peak}}$) and vascular flow [71, 72].

Respiratory training, and in particular inspiratory muscle training (IMT), is a type of training which aims to improve respiratory muscle endurance, through use of specific devices (the most used apply a resistive load or a threshold load about 30% of maximal inspiratory pressure) [73]. The rationale behind use of this type of training is the finding of changes in muscle fibers of diaphragm [74] and ventilatory abnormalities at cardiopulmonary exercise test in HF patients [52].

Several studies examined role of IMT in heart failure, showing improvement in ${\mathrm{VO}}_{\text{2peak}}$, maximal inspiratory pressure, QoL and other parameters [75, 76, 77, 78].

Combined with aerobic training, IMT showed additional benefits in serum biomarkers, such as C-reactive protein and NT-proBNP [78].

Functional electrical stimulation (FES) is a technique which uses surface electrodes to stimulate muscle activity. This technique represents an opportunity for patients with reduced mobility or who cannot tolerate exercise [79]. In 2013, a meta-analysis exploring the effects of FES in HF patients showed that, although with a lower effect size than other training modalities, FES significantly improved 6-Minute-Walking distance (6MWD) and ${\mathrm{VO}}_{\text{2peak}}$ compared to controls. In this view, FES could be used as a bridge-method to make patients able to perform conventional exercise training [80].

In Table 1 key elements of above-mentioned training modalities are shown. In recent years, the attitude to use the different training methods in combination with each other has become increasingly widespread. In a 2016 meta-analysis, Cornelis *et al*. [81] compared different training modalities, alone and in combination, to evaluate the effects on ${\mathrm{VO}}_{\text{2peak}}$, left ventricular ejection fraction (LVEF), left ventricular end-diastolic diameter (LVEDD) and QoL; no significant effects were found regarding CPET parameters, while there was a significant improvement in QoL in combined continuous and strength training, and a significant improvement in LVEF and LVEDD in interval training compared to continuous training

**Table 1.** **Key elements for exercise training modalities**.

	Starting protocol	Progression scheme	Main Effects
Moderate continuous training	10–15 minutes.	30 minutes.	Improve exercise tolerance, 6MWD, ${\mathrm{VO}}_{\text{2peak}}$, ${\mathrm{VE/VCO}}_{2}$; Improve cardiac output and diastolic function.
	Intensity: 40–50% of ${\mathrm{VO}}_{\text{2peak}}$.	Intensity: >60–70% of ${\mathrm{VO}}_{\text{2peak}}$.	
Interval training	High intensity: 4 minutes bouts at 90% of maximal exercise capacity, interspersed with 3 minutes recovery period.	Increase bouts intensity.	Improve exercise tolerance, 6MWD, ${\mathrm{VO}}_{\text{2peak}}$; Improve resting LVEF, LVEDD.
	5–10 minutes of warm–up and cool–down phases.		
	Exercise duration: 35–45 minutes.		
	Low intensity: Bout of 10 seconds and recovery period of 80 seconds.	Bout of 30 seconds and recovery period of 60 seconds.	
	Exercise duration: 5–10 minutes.	Exercise duration: 30 minutes.	
Strength training	5–10 repetitions.	15–25 repetitions.	Improve muscle mass; improve intramuscular co-ordination; increase resting LVEF.
	1–3 circuit each session.	1 circuit each session.	
	2–3 sessions/week.	2–3 sessions/week.	
	Intensity: <30%.	Intensity: 30–50%.	
	1-RM or Borg scale <12.	1-RM or Borg scale 12–15.	
Inspiratory muscle training	Use of threshold device at 20–30% of MIP for 15–30 minutes/day.	Readjust weekly. It is possible practice 2 session daily, 30 minutes each session, 7 days/week.	Improve respiratory muscle strength and endurance, 6MWD, ${\mathrm{VO}}_{\text{2peak}}$.
	5–6 days/week.		
Functional electrical stimulation	10 Hz frequency.		Improve 6MWD, exercise duration, ${\mathrm{VO}}_{\text{2peak}}$.
	20 second stimulation-20 second rest.		
	60 minutes/day.		
	7 days/week.		

1-RM, 1 repetition maximum; 6MWD, 6-Minute walking distance; LVEDD, left ventricular end diastolic diameter; LVEF, left ventricular ejection fraction; MIP, maximal inspiratory pressure; ${\mathrm{VO}}_{\text{2peak}}$, peak oxygen consumption; ${\mathrm{VE/VCO}}_{2}$, minute ventilation/carbon dioxide production.

In ARISTOS-HF trial [25], a new model of training, based on combined aerobic training/resistance training/inspiratory muscle training named ARIS (12 weeks, 3 times/week, 10 minutes/week, respectively) have been proposed. The idea behind this training modality was to improve functional capacity, which is impaired in HF patients, i.e., low aerobic capacity, reduced respiratory muscle function and pathological peripheral muscle strength. Although no statistically significant results were found in ARISTOS-HF trial, positive trend for increased ${\mathrm{VO}}_{\text{2peak}}$ and additional benefits in peak circulatory power (the product of ${\mathrm{VO}}_{\text{2peak}}$ and peak systolic blood pressure), LVEDD and QoL were shown in ARIS group; in particular, peak circulatory power showed to be a stronger predictor for cardiovascular events in HF patients [82, 83]. These findings allowed authors to encourage use of ARIS training in HF patients.

**Fig. 2.**
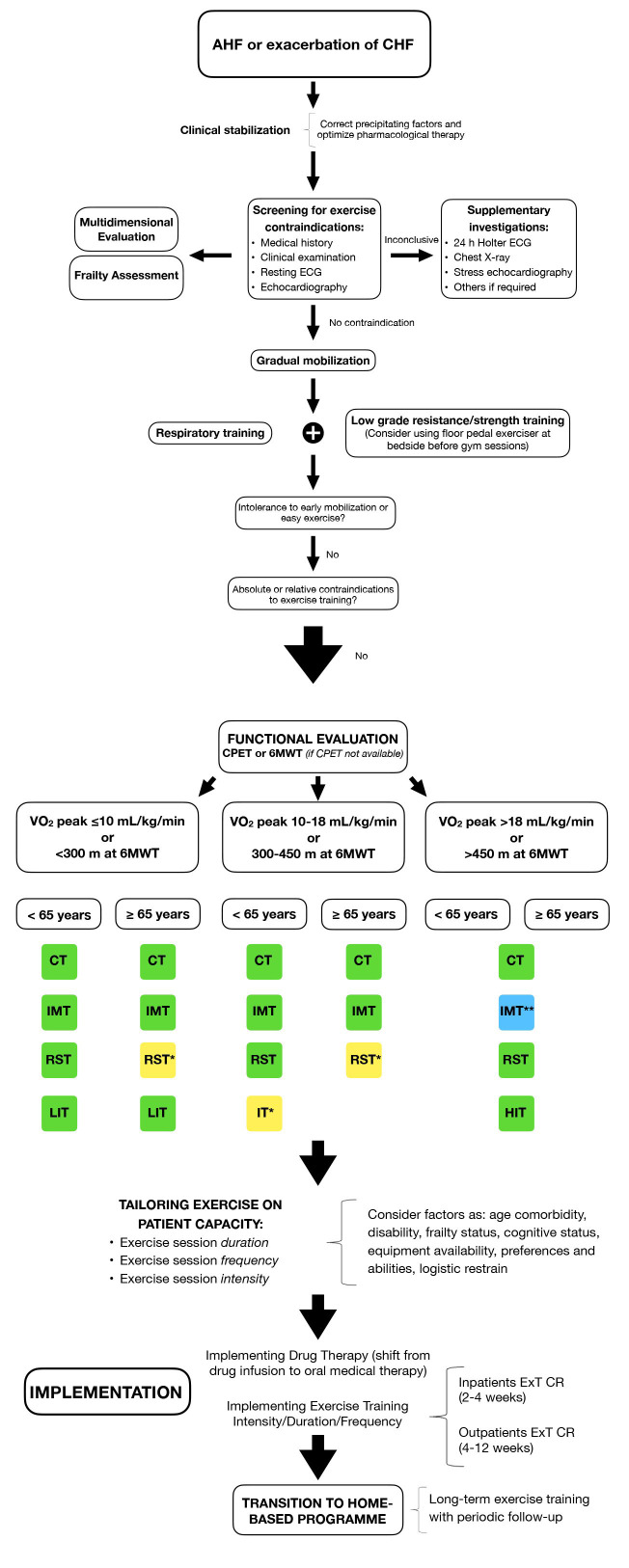
**Procedural algorithm of exercise training in Heart failure *(continue on next page)***. 6MWT, 6-Minute Walking Test; AHF, acute heart failure; CHF, chronic heart failure; CPET, cardiopulmonary exercise testing; CR, cardiac rehabilitation; CT, continuous training; ExT, Exercise Training; HIT, high intensity interval training; IMT, inspiratory muscle training; IT, interval training; LIT, low intensity interval training; RST, resistance strength training; VO_2_ peak, peak oxygen consumption. * (yellow): consider it in active lifestyle patients. ** (blue): consider it only if respiratory muscle weakness is present.

In Fig. 2, different rehabilitative modalities according to clinical stability and functional capacity, individual possibilities, and frailty status of HF patients have been proposed.

## 4. Prescription of Exercise Training in Patients with Heart Failure with Preserved Ejection Fraction (HFpEF) 

Although HFrEF and HFpEF are two different entities in terms of pathophysiology and background disease, they both present a common range of symptoms and reduced exercise tolerance is a hallmark. HFpEF is the most common form of HF in older population and in people with hypertension and other cardiovascular risk factors [14]. Surprisingly, few studies have been conducted to assess the beneficial effects of ET in HFpEF patients.

According to pathophysiological perspective, the main causes of exercise intolerance and reduction in ${\mathrm{VO}}_{\text{2peak}}$ in these patients are related either to cardiac or not cardiac patterns.

As for the former, in HFpEF patients undergoing exercise, alterations in LV stiffness and LV relaxation determine an increase in pulmonary capillary pression and, consequently, dyspnea and lower ${\mathrm{VO}}_{\text{2peak}}$ [84, 85].

Regarding peripheral mechanisms, it has been reported that arterial velocity pulse index (AVI), which is associated with ${\mathrm{VO}}_{\text{2peak}}$, is lower in HFpEF patients, suggesting importance of impaired vascular function in exercise intolerance genesis in this cohort [86]. Underlying mechanisms of reduced exercise tolerance in HFpEF patients are not fully elucidated; a reduced skeletal muscle hyperemia and a marked reduction in muscle mass replaced by an increase in intermuscular adipose tissue may play a key role [87, 88, 89].

In a randomized, controlled, single-blind study Kitzman *et al*. [82] enrolled 53 elderly patients with isolated HEpEF and evaluated them during 16 weeks of MCT; ${\mathrm{VO}}_{\text{2peak}}$ increased significantly in patients undergoing to exercise training (+2.3 ± 2.2 mL/kg/min, *p* = 0.0002), as well as 6-MWD (*p* = 0.0002) [90].

In a meta-analysis including 6 randomized controlled trials in which patients performed MCT, Pandey *et al*. [83] observed an improvement in ${\mathrm{VO}}_{\text{2peak}}$ and QoL, but not in echocardiographic parameters (E/A, deceleration time and ejection fraction) [91]. Different studies were concordant to the meta-analysis findings [92, 93], thus suggesting that improvements in exercise tolerance were independent from changes in systolic or diastolic function; while effects on peripheral mechanisms, as improved oxygen extraction by skeletal muscles, could be implicated.

In 2015, Angadi *et al*. [86] compared HIIT vs. MCT exercise in HFpEF patients reporting an increase in ${\mathrm{VO}}_{\text{2peak}}$ (from 19.2 ± 5.2 to 21.0 ± 5.2 mL/kg/min; *p* = 0.04) and a statistically significant improvement in diastolic markers (E and deceleration time, *p* = 0.02) in patients undergoing 4 weeks HIIT, while no significant changes were observed in patients undergoing MCT. Although sample size was limited and designed a short follow-up period (4 weeks), these findings paved the way for considering short-term HIIT protocol in HFpEF patients [94].

Moreover, Donelli da Silveira *et al*. [95] in randomized clinical trial demonstrated the superiority of HIIT vs. MCT in HFpEF patients after 12 weeks exercise program. In particular, this trial showed that the increase in ${\mathrm{VO}}_{\text{2peak}}$ is two times higher in the HIIT compared to MCT group [3.5 (3.1 to 4.0) vs. 1.9 (1.2 to 2.5) mL/kg/min, *p *< 0.001]; while similar improvements in diastolic function and QoL have been reached through both training modalities [95].

These findings highlight that HIIT is more effective in HFpEF compared to MCT, probably due to the improvement in diastolic dysfunction; however, conflicting results have been reported [96].

In a recent meta-analysis, IMT was effective in HFpEF patients in improving 6MWD (mean difference 83.97 meters, 95% CI, 59.18–108.76; *p *< 0.0001) and ${\mathrm{VO}}_{\text{2peak}}$ (mean difference 2.82 mL/kg/min, 95% CI, 1.90–3.74; *p *< 0.0001) [97].

Although older age and poor effort tolerance could make exercise difficult to perform, the proven efficacy in different trials and the shortage of therapeutic options for this condition strongly suggest using these exercise training protocol in HFpEF patients.

## 5. Exercise Training in HF Elderly Patients 

Prevalence of HF rise to approximately 10% among people >70 years old [2, 6]. In addition, regardless of comorbidities, a reduction in ${\mathrm{VO}}_{\text{2peak}}$ from 45 mL/kg/min in young people (25 years old) to 25 mL/kg/min in older people (75 years old) have been described [98].

Practicing ET is quite difficult in HF elderly patients although they represent the majority of HF cohort. In Fig. 2, a procedural algorithm exploring all rehabilitative modalities according to clinical stability and functional capacity, individual possibilities, and frailty status of HF patients has been proposed. Notably, elderly HF patients should be carefully evaluated for tailoring exercise session according to their peculiar characteristics (disability, frailty, cognitive impairment, falls risk, sarcopenia, visual and ear impairment, etc.).

ET has been largely investigated in older patients [99, 100, 101]. Austin* et al*. [99] enrolled 200 patients >60 years old with NYHA class II–III and randomized to ET group or usual care: patients performed aerobic endurance training and low resistance strength training 2.5 hours for session, 2/week for 8 weeks, and afterwards other 16 weeks exercise sessions consisting of 1 hour/week. After 24 weeks training, ET group showed a significant improvement in functional capacity [6MWD increases significantly by 16% in ET group (from 275.5 ± 21.4 meters to 320.4 ± 21.9 meters; *p *< 0.001)], in functional status [NYHA class (from 2.44 to 2.01)] and QoL, while hospital admissions were fewer and lasted less compared to usual care controls.

More recently, Antonicelli *et al*. [100] investigated ET effect in 343 older HF patients (<70 years, mean age 76.90 ± 5.67): patients performed endurance training 3 times/week for 3 months in hospital settings and next 3 months in home-monitored settings; 6MWD improved from 299 ± 120 meters to 394.1 ± 123.6 meters after 6 months in exercise group (*p *< 0.001), all-cause hospitalizations adjusted for clinical covariates reduced by 44.2% (B = 0.558, 95% CI, 0.326–0.954, *p* = 0.033) and was shown improvement in QoL (28.6 ± 12.3 vs. 44.5 ± 12.3, *p* = 0.001). Furthermore, it was found a significant reduction in NT-proBNP plasma levels in ET group from 1236 to 440 pg/mL (*p *< 0.001), while this level increased in control group.

In a cohort of 40 postinfarction older patients, Giallauria *et al*. [102] reported that 3-month ET program was associated to a reduction in NT-pro-BNP levels (from 1446 ± 475 to 435 ± 251 pg/mL, *p *< 0.001) and an overall improvement of exercise capacity, without LV remodeling and with improvement in early LV filling. Interestingly, an inverse correlation between changes in NT-pro-BNP levels and in ${\mathrm{VO}}_{\text{2peak}}$ (r = –0.67, *p *< 0.01), E-wave (r = –0.42, *p *< 0.01) and E/A ratio (r = –0.60, *p *< 0.01) have been reported; suggesting that ET can exert its beneficial effects by improving myocardial efficiency with no detrimental effects even in elderly patients.

Although aerobic endurance training has been more investigated in elderly HF patients, skeletal muscle wasting is a precipitating factor in clinical conditions in these patients, causing increase in type II muscular fibers and consequently an earlier shift to anaerobic metabolism and fatigue onset [103, 104].

Reduced ${\mathrm{VO}}_{\text{2peak}}$ and lower exercise time were found associated with sarcopenia in type I muscular fiber area was predictive of changes in 6M [105]. Pu *et al*. [101] investigated progressive resistance training in 16 HF patients (100% women, mean age 77 ± 6 years) compared to non-HF individuals with comparable aerobic capacity. All patients performed 60 minutes session, 3 session/week, for 10 weeks. After 10 weeks, exercise group showed a significant improvement in 6MWD (+49 ± 14 meters; *p *< 0.03) and muscle strength (33.5 ± 7.3% increase on leg press and 68.0 ± 13.2% on knee extension); notably, change WD (r = 0.612; *p* = 0.026).

Combined muscle strength and aerobic training programs have been proved to increase ${\mathrm{VO}}_{\text{2peak}}$ and to improve other CPET indexes [106]. A recent meta-analysis investigated the ET effects in older patients with HF and evaluated relationship between training modalities and efficacy [107]. ET improved QoL (effect size = –0.69; *p *< 0.001), aerobic capacity (measured as 6MWD, effect size = 0.47; *p* = 0.002) and cardiac function (measured as LVEF, effect size = 0.91; *p* = 0.001). In addition, resistance training had greatest effect on aerobic capacity, while aerobic training had greatest effect on cardiac function. Duration of intervention, duration of single session and weekly frequency showed to have no predictive influence on aerobic capacity and cardiac function adaptation.

Although it would be desirable to have more trials, data available suggest that ET have similar benefits in older HF patients compared to younger cohort. Combined strength and aerobic training should be recommended to prevent wasting syndrome in older patients in addition to effects on aerobic capacity [108, 109, 110].

## 6. Low Intensity Exercise Training for Frail Patients with Heart Failure

Patients with poorer clinical condition are often excluded by most trials; although in these specific patients, low-intensity exercise have major impact on quality of life favorably changing perspectives for daily life activities [111]. In HF patients, ET exerts beneficial effects not only improving physical performance, but also restoring basic abilities, particularly in patients with poorest conditions. Early gradual mobilization in patients with cachexia or after recent acute event is strongly recommended [58]. These movements are performed using only resistance opposed by their own weight, aiming at increasing strength, at improving coordination, respiratory capacity. These protocols should be performed at low intensity, with gradual increase according to patient’ perceived exertion.

In the REHAB-HF pilot study [112], 27 patients older than 60 years which experienced acute decompensated heart failure were assigned to an intervention group performing multi-domain rehabilitation which included combined strength (sit to stand), balance (stand and reach), endurance (continuous walking) and mobility (dynamic start and stop) exercises compared to control group. Starting objectives were rise from chair using hands, stand with feet apart and walk for at least 10 minutes; at the last level of intensity patients were able to sit to stand behind chair with arms across chest, stand in semi-tandem and walk for 30 minutes quickly changing direction. The primary outcome was change in Short Physical Performance Battery (SPPB) test, which assess speed over 4 meters, time to complete 5 chair rises and standing balance: after 3 months SPPB score in intervention group increased from 4.8 ± 2.8 to 6.9 ± 3.0 units compared to increase from 6.0 ± 3.0 to 6.8 ± 3.3 units in control group; moreover, it was shown an increase in 6MWD from 170 ± 83 meters to 232 ± 113 meters in exercise group. Also, inversely correlation in SPPB score with 6 months all cause rehospitalizations (–0.60; *p *< 0.01) was observed. Of note, SPPB is a very common test among geriatricians for great predictive power, and could be easily adopted as outcome measure in frail elderly patients when cardiopulmonary exercise stress testing is not feasible or available [113]; and the adoption of other outcomes measures to evaluate the effects of exercise training in specific cohorts of patients (i.e., frail elderly patients) should be encouraged [8].

**Fig. 3.**
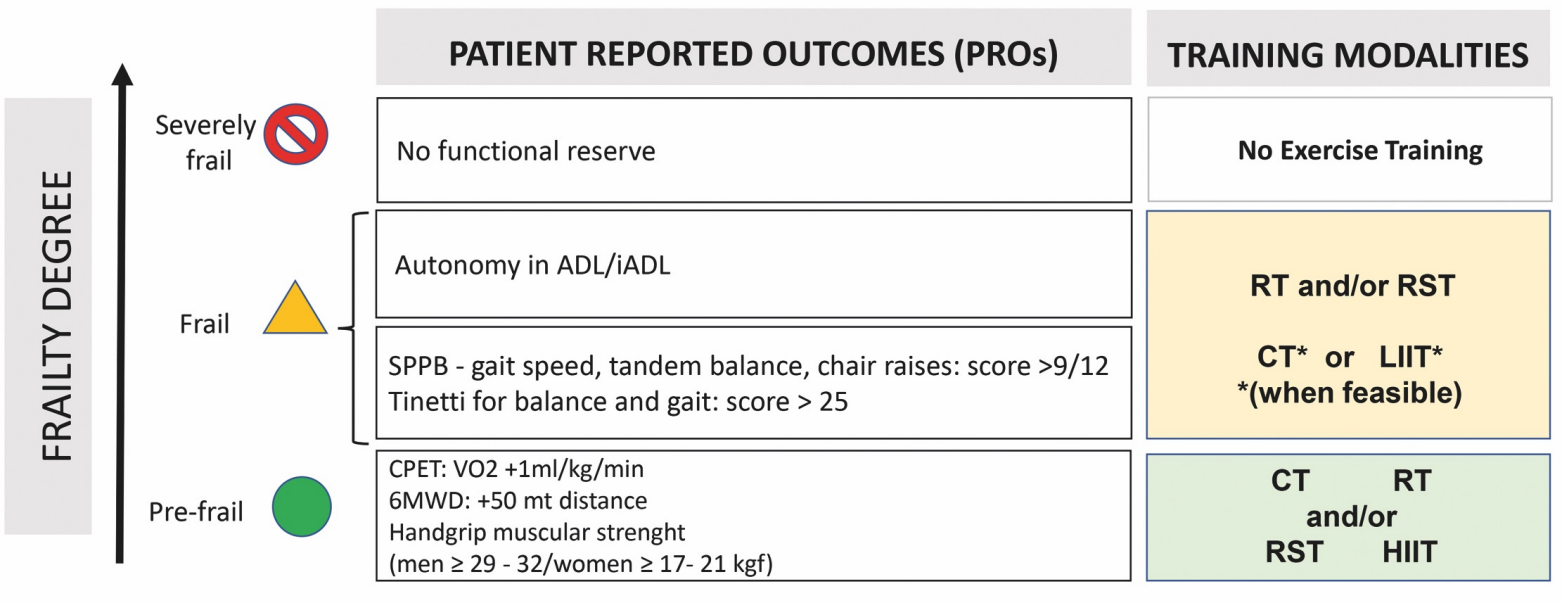
**Patients Reported Outcomes (PROs) and training modalities according to Frailty degree**. 6MWD, 6-minute walking distance; ADL, activities of daily living and iADL, instrumental activities of daily living; CPET, cardio-pulmonary exercise test; CT, continuous training; HIIT, high intensity interval training; LIIT, low intensity interval training; RST, resistance/strength training; RT, respiratory training; SPPB, short physical performance battery.

Therefore, in elderly HF patients, exercise prescription must be tailored on patient’s status and reach patient’s individualized targets (Patients Reported Outcomes, PROs) (Fig. 3). When feasible, undergoing CPET is considered the gold standard for evaluating functional capacity; otherwise 6MWT distance should be considered (Figs. 2,3). Both tests are strongly related to patient’ outcome. In elderly HF patients, functional capacity progressively worsens; and patients might not be able to complete these tests in several conditions such as pre-frailty, frailty, comorbidity, physical disability, polypharmacy, cognitive status, sedentary behavior, work abilities, etc. In these patients, geriatric multidimensional evaluation is mandatory (Figs. 2,3).

## 7. Impact of Exercise Training on Mortality

Despite the rationale and biological plausibility in favor of ET in HF, trials often fail to demonstrate a reduction in mortality. The large HF-ACTION trial failed to show a reduction in all-cause (HR = 0.96 [95% CI, 0.79–1.17]; *p* = 0.70) and cardiovascular mortality (HR = 0.92 [95% CI, 0.83–1.03]; *p* = 0.14) in exercise training group vs. usual care group in primary analysis, and only after supplementary analyses adjusting for highly prognostic baseline characteristics a statistically significant reduction in all–cause mortality was found (HR = 0.89 [95% CI, 0.81–0.99]; *p* = 0.03) [22].

The ExTraMATCH meta-analysis aimed at assessing exercise training effect on mortality in 801 HF patients, 396 assigned to exercise group and 406 assigned to control group; in exercise training group, mortality resulted significantly lower (log rank χ^2^ = 5.9, *p* = 0.015) [114].

More recently, in order to include more patients and to more thoroughly evaluate the effects of exercise training in Heart Failure, the ExTraMATCH II meta-analysis was conducted [18]; although no reduction in mortality and hospitalizations was observed, a statistically significant improvement in exercise capacity and QoL was found (mean improvement at 6-MWT 21 m, 95% CI, 1.57–40.4 m, *p* = 0.034; mean difference at Minnesota Living with Heart Failure Questionnaire score –5.94, 95% CI, –1.0 to –10.9, *p* = 0.018) and positive trend in ${\mathrm{VO}}_{\text{2peak}}$ was observed (1.01 mL/kg/min, 95% CI, –0.42 to 2.44 mL/kg/min; *p* = 0.168).

A recent meta-analysis reported that an exercise-based cardiac rehabilitation program had no impact on mortality in first 12 months but obtaining additional data by contacting the study authors resulted in a reduction in all-cause mortality at a follow-up of more than 12 months in patients who performed ET compared to control group (intervention 244/1418 (17.2%) vs. control 280/1427 (19.6%) events): RR 0.88, 95% CI, 0.75–1.02; *p* = 0.09) [115].

Even if mortality data do not lead to univocal results, the improvement in ${\mathrm{VO}}_{\text{2peak}}$ and 6MWD could be reliable surrogate parameters for assessing exercise effect on final outcomes in HF patients.

## 8. SARS CoV-2: Heart Failure Rehabilitation during Pandemic

The severe acute respiratory syndrome coronavirus 2 (SARS CoV-2) in 2020 started a pandemic which created major difficulties for Health Systems of worldwide countries in recent years and risks changing health care in the future.

SARS CoV-2 infection and its disease “COVID-19”, demonstrated more severe course and higher mortality in patients with cardiovascular comorbidities [116, 117]. The discovery that SARS CoV-2 enters human cells through angiotensin-converting enzyme 2 (ACE2) receptor created several concerns particularly in patients treated with renin angiotensin system (RAS) inhibitors (drugs largely used in cardiovascular diseases and especially HF) that in early phases where deemed to promote COVID-19 disease [118]. Notably, several studies demonstrated that use of ACE inhibitors (ACEI) or angiotensin-receptor blockers (ARBs) is not associated with risk of more severe COVID-19 disease [119, 120].

Although COVID-19 is prevalently a respiratory disease, systemic involvement (cardiovascular, gastrointestinal, neurological, renal, thromboembolic, etc.) has been clearly documented [121, 122, 123, 124, 125]. Cardiovascular manifestations may be secondary to lung disease, which causes respiratory failure, hypoxia and increasing cardiac workload: however also other mechanisms have been shown, as coronary microvascular damage [126] and direct cardiac injury due to virus capacity to directly infects human cardiomyocytes, causing increase of cardiac troponins [127, 128, 129].

Therefore, patients who already present cardiovascular comorbidities should be particularly beware for risk of a SARS CoV-2 infection. This is particularly valid for those suffering from HF: Matsushita *et al*. [130] randomized 889 French patients with previous acute coronary syndrome, dividing them in a reduced LVEF group (EF <40%, n = 91) and moderated reduced and preserved LVEF group (EF ≥40%, n = 798); higher incidence of COVID-19 related hospitalization or death resulted in reduced LVEF group (9% vs. 1%, *p *< 0.001), regardless discontinuation of ACEI or ARBs. Moreover, it has been supposed that COVID-19 through proinflammatory cytokines activation could unmask asymptomatic HFpEF or contribute to progression in patients with already known disease [131].

In addition to the direct damage caused by COVID-19, reorganizations of healthcare resources to deal with the pandemic emergency has also caused difficulties for patients with HF. Many countries ordered a lockdown in early stages of pandemic trying to limit infections, with obvious limitations to outdoor exercise training and to cardiac rehabilitation programs participation for cardiac patients.

Cunha *et al*. [132] evaluated lockdown impact on physical activities and vital sign in HF patients with an implantable cardioverter defibrillator (ICD) or cardiac resynchronization device (CRT) highlighting marked reduction in physical activity, especially in patients with performed low exercise before lockdown: this may lead to worsening of clinical status of these patients in future, increasing hospitalization and mortality.

Worldwide, scientific societies proposed different modalities, either in telemedicine or through protocols to be implemented in hospital setting, trying to guarantee cardiac rehabilitation programs continuation during pandemic [133, 134, 135, 136]. The effectiveness of solutions implemented will certainly be one of most important challenges that healthcare systems will have to face in this century to ensure survival and quality of life in patients with cardiovascular diseases.

## 9. Exercise Training Limitations

Although ET programs are strongly recommended by Guidelines, recent RCT are showing less significant results regarding ET effects in heart failure. One explanation is that in previous trials, particularly those prior to 1990s, many patients were not treated on OMT, which includes beta-blockers, aldosterone antagonists or angiotensin receptor Neprilysin inhibitors.

The impossibility of being performed in the most fragile patients and in which exercise would represent a risk than a therapeutic alternative is one of the flaws of exercise training protocols. Cardiac and non-cardiac diseases in which exercise is contraindicate have been defined in a consensus document of the Heart Failure Association and the European Association for Cardiovascular Prevention and Rehabilitation [58]. However, this issue could be limited in certain patients through use of functional electrical stimulation (FES) which is also suitable for patients with reduced mobility [79].

Unfortunately, HF women are often denied to cardiac rehabilitation programs [137, 138]; specific CR programs specifically designed for women are eagerly awaited.

Finally, the poor patient’s adherence to training programs is likely the most important limiting factor for the lack of benefit observed in trials [139]. The HF-ACTION trial, a larger multicenter RCT which aimed to investigate effects of exercise training on mortality and safety, failed to meet expectations, probably because patient’s participation in training programs was on average 1.8 times/week compared to 3 times/week foreseen by protocol [22].

In EXERT trial HF patients performed aerobic and resistance training for 30 minutes 2 times/week for at least 9 months, of which first 3 months under supervision; the investigator found a reduction in number of training sessions when performed at home [140].

Therefore, trying to improve patients’ adherence to training program could be the best way to improve its effectiveness. It is mandatory to consider that adherence to training program is always more difficult than pharmacological therapy, as it requires more dedicated time.

Furthermore, adherence is affected by patient-related factors, such as severity of symptoms, age, sex, comorbidities and socioeconomic status, and by factors related to the rehabilitation center, such as logistics and availability of physician [141, 142, 143, 144, 145, 146].

To improve patient’s adherence, it is important to be very clear in explaining the number of training sessions, the effort to be made during exercise and its duration and, above all, the exercise modalities. Supervised and encouraged exercise is the best way to keep patients motivated [139], so it is advisable to increase duration of supervised exercise phase during trials.

Initiatives aiming at encouraging patients’ adherence to cardiac rehabilitation, such as TAKEheart (Training Awareness Knowledge Engagement) by AHRQ (Agency for Healthcare Research and Quality, more information to https://takeheart.ahrq.gov) pave the way for improving attendance to exercise-based cardiac rehabilitation programs.

Promoting exercise group sessions and psychological support for patient without family/friends support could represent a valid strategy for improving adherence to training programs. This modality has been successfully used in other conditions (i.e., cancer) [147], but data are still lacking for cardiovascular diseases. However, during COVID-19 pandemic, this option does not seem preferable at present and it is discouraged by Healthcare stakeholders [133, 134, 135, 136].

Finally, it is important to ensure that training benefits are clear to patients; the self-efficacy technique was investigated to keep compliance high [148, 149]. Questionnaires and diaries filled in by patients can be used to monitor progress of training protocol.

## 10. Conclusions

Exercise training is widely recognized as an evidence-based adjunct treatment modality for patients with HF, and growing evidence is emerging among elderly patients with HF. Exercise training exerts both central and peripheral adaptations that clinically translate into anti-remodeling effects, increased functional capacity and reduced morbidity and mortality. Ideally, exercise training programs should be prescribed in a patient-tailored approach, particularly in frail elderly patients with HF. Increasing long-term adherence and reaching the frailest patients are challenging goals for future initiatives in the field.
